# Perspectives of healthcare workers in South Africa on COVID-19 vaccination passports

**DOI:** 10.4102/hsag.v27i0.1823

**Published:** 2022-04-25

**Authors:** Claudia J. Jansen van Vuuren, Juan M. Jansen van Vuuren

**Affiliations:** 1 Centre for the AIDS Programme of Research in South Africa (CAPRISA), Nelson R Mandela School of Medicine, University of KwaZulu-Natal, Durban, South Africa; 2 School of Clinical Medicine, University of KwaZulu-Natal, Durban, South Africa; 3 Department of Internal Medicine, Grey’s Hospital, Pietermaritzburg, South Africa

**Keywords:** COVID-19, vaccine hesitancy, healthcare workers, vaccine passports, ethics

## Abstract

**Background:**

Following the rollout of several effective vaccines against coronavirus disease 2019 (COVID-19), many countries have introduced vaccination passports or certificates as a means of certifying that an individual has been vaccinated against, is immune to, or is presently uninfected with COVID-19. An extensive ethical debate has ensued.

**Aim:**

To determine the perspectives of South African healthcare workers (HCWs) on the implementation of COVID-19 vaccination passports (C19VPs) in South Africa (SA).

**Setting:**

Healthcare workers working in various fields and practice settings throughout SA were invited to complete an online questionnaire.

**Methods:**

An online questionnaire was distributed using convenience sampling via social media platforms to HCWs over a 1-month period, collecting demographic details and responses to 8 Likert-type items regarding agreement with C19VPs, ethical issues and feasibility. Each item was graded from 1 (strongly disagree) to 5 (strongly agree), with grouping of 4 of the 8 items exploring a common theme of C19VPs being a good idea, constituting a score out of 20. Non-parametric tests were performed to determine differences in responses between groups.

**Results:**

One thousand HCWs responded to the survey and fulfilled inclusion criteria. The majority (83.2%) of respondents were medical practitioners (MPs). Overall, most (73.5%) respondents agreed that C19VPs are a good idea. Older respondents agreed more strongly than younger respondents (medians 18 and 17, respectively, *p* = 0.001), and respondents in private practice agreed more strongly than those in state practice (medians 18 and 16, respectively, *p* = 0.042). The median response was neutral (3) in response to the ethics of C19VPs considering variations in vaccine access and tending towards disagreement (2.5) in disadvantaging poorer people. Most respondents disagreed that vaccine hesitancy would make C19VPs unethical, and responses from provinces with the highest vaccination proportions disagreed more than others with lower vaccination proportion (median 2 compared with 3, *p* < 0.001). There was uncertainty about the feasibility of C19VPs in SA, with older HCWs, non-students, senior MPs and those who thought C19VPs are a good idea being more likely to consider them feasible.

**Conclusion:**

The perspectives of HCWs, mainly MPs, about C19VPs in SA were obtained. Further research should focus on vaccine hesitancy and its factors in HCWs and the effect of C19VPs on restrictions, reduction in transmission and benefits on economies and mental health.

**Contribution:**

To the authors’ knowledge, this is the first survey data published on the perspectives of South African HCWs on C19VPs in the country. Healthcare workers are trusted influencers of vaccination decisions, and their opinion on vaccination certificates may also influence the South African public’s perception and acceptance thereof.

## Background

Following the global rollout of coronavirus disease 2019 (COVID-19) vaccines, many countries began to introduce so-called ‘vaccination passports’, or immunity licences, as a means of certifying that an individual has been vaccinated against, is immune to, or is presently uninfected with COVID-19 (Phelan 2020). The introduction of this form of certification has triggered an ethical debate around access to and acceptability of vaccines, vaccine hesitancy, concerns over privacy, actual and perceived human rights violations and the creation of ‘perverse incentives’ for individuals to seek out infection (Cellan-Jones 2021; Dye & Mills 2021; Johnson, Fraser & Sato 2021; Phelan 2020).

Practical implications of implementing immunity-based certificates have been raised, for example, where an individual may falsely be declared immune due to inaccurate serological tests (Persad & Emanuel 2020). This would facilitate community transmission due to an individual being able to engage in higher-risk activities based on their ‘confirmed’ immune status (Persad & Emanuel 2020). The benefits of the certification may also encourage forgery of passports, or even lead to fraud by healthcare workers (HCWs) and testing facilities (Persad & Emanuel 2020).

Conversely, ethicists have highlighted the problems with unnecessarily restricting movement in individuals considered to be immune, as well as highlighting benefits such as a resumption of pre-pandemic normality, lifting of restrictions on free movement (i.e. an end to lockdowns), reducing social harms caused by unemployment and isolation, allowing the reopening of small businesses and restaurants and enabling people to attend cultural, worship and sporting events in person (Brown et al. 2021, De Miguel Beriain & Rueda 2020; Persad & Emanuel 2020).

Despite this ongoing debate, several countries have introduced COVID-19 vaccination passports (C19VPs) due to the public health benefits ascribed. Most of these are stored digitally, can be retrieved using unique QR codes and have allowed for the easing of some restrictions (European Commission 2020; Johnson et al. 2021; National Health Service 2021; Whyte 2021). On 08 October 2021, the South African COVID-19 Vaccine Certificate System was launched, which links to the Electronic Vaccination Data System (EVDS), producing digital proof of vaccination, which President Cyril Ramaphosa has said ‘can be used to facilitate travel, [for] access to establishments and gatherings, and other forms of activity that require proof of vaccination status’ (Daniel 2021).

In SA, there is a legal framework for the introduction of a mandatory vaccination policy in the workplace, with the *National Health Act* No. 61 of 2003 considering the rights of people ‘to an environment that is not harmful to their health or well-being’ (South Africa 2003), a right which is also enshrined in section 24(a) of the SA Constitution (Constitution of South Africa 1996). A directive from the country’s Minister of Employment and Labour in June 2021 gave instructions to employers about their duties to employees regarding making vaccines mandatory. Employers’ decisions on mandatory vaccination must take into account the requirements of the *Occupational Health and Safety Act* No. 85 of 1993 (South Africa 1993), as well as the operational requirements of the workplace (Dhai 2021). It has been proposed that it could be considered ‘reasonable and justifiable’ to mandate vaccination in certain groups of workers to uphold the rights of all people to a safe environment, even where this may involve limiting the individual’s rights to freedom of religion, belief and opinion (Dhai 2021). Multiple South African organisations have since introduced mandatory vaccine policies (Abdool Karim 2021).

With these factors in mind, the researchers sought to obtain the perspectives of HCWs in SA on the concept and implementation of C19VPs in SA, as one of the groups of workers most exposed to COVID-19 and who may face vaccine mandates in the workplace.

## Methods

### Study design

A cross-sectional, quantitative study design was followed, inviting HCWs to complete a single survey to determine their knowledge, attitudes, practices and beliefs (KAPB) on C19VPs.

### Setting

Healthcare workers working in various fields and practice settings throughout SA were invited to complete an online questionnaire.

### Sampling

Non-probabilistic, convenience sampling was used. With an estimated population of South African HCWs of 650 000 (Kerr & Thornton 2020), with a 95% confidence level and a 5% margin of error, the target sample size was calculated as 384. Respondents were invited to participate in the survey between 19 July 2021 and 22 August 2021, with the link to the online Google Forms questionnaire being distributed via word of mouth, social media (South African HCW Facebook Groups and LinkedIn networks) and bulk email distribution by the South African Medical Association (SAMA) to its members. To complete the survey, respondents had to be currently employed or employable as an HCW in SA, registered or registrable with a professional healthcare body or regulatory authority in SA, adults aged 18 years or older, in the private, state or mixed practice setting and included all levels of practice. Response forms were excluded from analysis if they did not fulfil these criteria.

### Data collection

The following variables were recorded: age group, SA residency status (citizen, permanent resident or visa holder), professional or regulatory authority (Health Professions Council of South Africa [HPCSA], South African Nursing Council [SANC], among others), employment status (employed, retired or studying), healthcare sector (private, state/public, or both), province, professional group (per HPCSA or SANC categories), profession, and level of medical practice in the case of medical practitioners (MPs).

**FIGURE 1:**
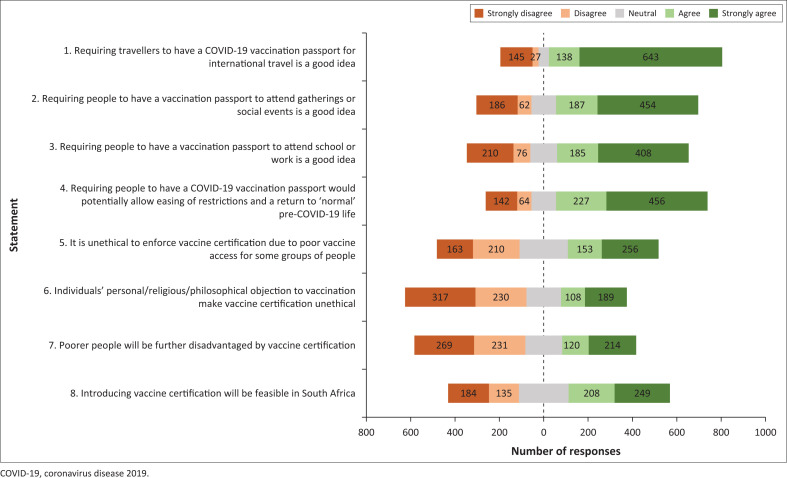
Responses to Likert-type items.

Following these demographic details, respondents were asked for their degree of agreement with 8 Likert-type statements, with 5 possible responses graded from 1 (‘Strongly Disagree’) to 5 (‘Strongly Agree’), which are shown in Figure 1. Likert items were designed to determine the range of HCW perspectives on the possible application of C19VPs to different settings (items 1–3), possible effect on restrictions (item 4), ethical concerns raised by their implementation (items 5–7) and on the feasibility of their implementation (item 8).

### Data analysis

Likert items 1 through 4 represented similar statements and were grouped into a Likert scale (Cronbach’s alpha = 0.94), with responses to the 4 statements thus contributing to a maximum of 20 points (4–7 = strongly disagree; 8–11 = disagree; 12 = neutral; 13–16 = agree; 17–20 = strongly agree), representing the statement ‘C19VPs are a good idea’. Statements 5 to 7 related to ethical issues surrounding C19VPs but were not grouped as they explored dissimilar concepts.

Simple descriptive statistics were calculated on the Likert-type items, with the median and interquartile range (IQR) being the most appropriate measures of central tendency and spread in this type of data (El Omda & Sergent 2021).

The data did not follow a normal distribution; thus non-parametric tests were performed to find differences in responses between groups. The Kruskal-Wallis test was performed to determine whether there were differences in the mean ranks of responses between at least one pair of groups when there were three or more groups being compared; with the null hypothesis being that the mean ranks of all groups are the same, degrees of freedom (*df*) equal to a number of groups-1, and a *p* value of < 0.05 resulting in rejection of the null hypothesis. In samples where the shape and scale of the distribution is the same among groups, a Kruskal-Wallis test with *p* < 0.05 indicates a difference in medians. In the cases of *p* < 0.05 for the Kruskal-Wallis test, Dunn’s post-hoc tests were then performed on each pair of groups, with *p*_bonf_ < 0.05 (p value adjusted due to multiple groups with Bonferroni correction) indicating a statistically significant difference between two of the groups assessed in the Kruskal-Wallis test.

The Mann-Whitney *U* test was used to compare whether there was a difference in the responses when comparing only two groups. The null hypothesis for each test was that the distribution in responses between the two groups was equal, with *p* < 0.05 resulting in rejection of the null hypothesis. As with the Kruskal-Wallis, when the shape of the distribution of responses between two groups is similar, a statistically significant Mann-Whitney *U* test indicates a difference in medians.

Grouping was performed for certain demographic details, such as age and profession, in addition to the grouping of provinces according to their proportion of the population vaccinated at the time of the survey, with data sourced from Covid19SA.org, a collaboration between the University of the Witwatersrand and the National Research Foundation’s iThemba LABS (COVID-19 SA 2020). These groups are outlined in more detail in the ‘Results’ section below.

Ordinal logistic regression analyses were performed between responses to different Likert items to generate response outcome probability prediction. A McFadden’s pseudo R-square of 0.2–0.4 was considered an excellent fit. Goodness of fit indicators (Pearson residuals chi-square and deviance residuals chi-square) *p* values of < 0.05 indicated a lack-of-fit. SigmaXL version 9.03 MAC was utilised for ordinal logistic regression analyses, while JASP version 0.14.1.0 was used for the aforementioned analyses.

### Ethical considerations

The protocol for this study was reviewed and approved by the uMgungundlovu Health Ethics Review Board on 06 May 2021 (UHERB 003/2021) and was approved by all nine provincial Departments of Health following review via the National Health Research Database. An information sheet was made available to participants and informed consent was required before continuing with the survey.

## Results

There were 1053 responses to the survey, of which 1000 met the inclusion criteria (see ‘Sampling’ above). Of the 53 sets of responses excluded from analysis, 36 reported not to be HCWs, and 17 reported they were not currently employed or studying in SA. The demographic details of the respondents are shown in Tables 1 to 3.

**TABLE 1:** Demographics.

Characteristics	*n*	%
**Age**		
18 to 24	37	3.70
25 to 34	389	38.90
35 to 44	163	16.30
45 to 54	161	16.10
55 to 64	127	12.70
Over 65	123	12.30
**Employment status**		
Employed	902	90.20
Retired	56	5.60
Studying	42	4.20
**SA residency**		
SA citizen	964	96.40
Permanent resident or visa	36	3.60
**Sector†**		
Private sector only	400	44.35
State/public sector only	398	44.12
Both public and private sectors	104	11.53
**Province†**		
Gauteng (GP)	330	36.59
KwaZulu-Natal (KZN)	249	27.61
Western Cape (WC)	180	19.96
Eastern Cape (EC)	52	5.76
Free State (FS)	26	2.88
Mpumalanga (MP)	23	2.55
North West (NW)	22	2.44
Limpopo (LP)	11	1.22
Northern Cape (NC)	9	1.00

† , recorded only for employed healthcare workers.

**TABLE 2:** Respondents’ professions.

Professions	*n*	%
**Dietetics and nutrition**	**1**	**0.1**
Dietician	1	0.1
**Emergency care**	**14**	**1.4**
Emergency Care Assistant	1	0.1
Emergency Care Practitioner	12	1.2
Emergency Care Technician	1	0.1
**Medical and dental (and medical science)**	**838**	**83.8**
Clinical Associate	1	0.1
Dentist	4	0.4
Health Assistant	1	0.1
Medical Practitioner	832	83.2
**Medical technology**	**2**	**0.2**
Medical Technologist	1	0.1
Supplementary Laboratory Assistant	1	0.1
**Nursing**	**69**	**6.9**
Enrolled Nurse	6	0.6
Enrolled Nursing Assistant	2	0.2
Registered Midwife	4	0.4
Registered Nurse	57	5.7
**Occupational therapy, medical orthotics, prosthetics and arts therapy**	**7**	**0.7**
Occupational Therapist	7	0.7
**Optometry & dispensing opticians**	**1**	**0.1**
Optometrist	1	0.1
**Pharmacy**	**10**	**1.0**
Pharmacist	9	0.9
Pharmacist’s Assistant	1	0.1
**Physiotherapy, podiatry and biokinetics**	**28**	**2.8**
Biokineticist	1	0.1
Physiotherapist	27	2.7
**Psychology**	**10**	**1.0**
Psychologist	10	1.0
**Radiography and clinical technology**	**10**	**1.0**
Clinical Technologist	6	0.6
Radiographer	4	0.4
**Speech language and hearing professions**	**10**	**1.0**
Audiologist	9	0.9
Speech Therapist and Audiologist	1	0.1

**TABLE 3:** Medical practitioner level of practice.

Levels of MP	*n*	%
MO	243	29.21
Consultant	214	25.72
Registrar	65	7.81
Intern	65	7.81
GP	55	6.61
Retired	49	5.89
Other	42	5.05
Student	39	4.69
CSMO	33	3.97
HOD	27	3.25

MP, medical practitioner; MO, medical officer; GP, General Practitioner/Family Physician; CSMO, Community Service Medical Officer; HOD, Head of Department.

Table 4 shows the median, first quartile (Q_1_) and third quartile (Q_3_) for Likert responses, including the responses for grouped statements 1–4. The statements and responses are illustrated in Figure 1.

**TABLE 4:** Median and quartiles responses for each statement.

Statement	Median	First quartile (Q_1_)	First quartile (Q_3_)
1	5	4	5
2	4	3	5
3	4	2	5
4	4	3	5
1–4	17	12	20
5	3	2	5
6	2	1	4
7	2.5	1	4
8	3	2	4

### Statements 1–4: Are COVID-19 vaccination passports a good idea?

Most respondents agreed that C19VPs are a good idea, with 73.5% of responses yielding a score of 13 or more for the grouped statements 1–4.

There was a difference (*p* = 0.012) in responses to these statements when comparing respondents in different age brackets (see Table 5), with similar distribution plots in all age brackets, indicating a difference in the median of at least two groups. Dunn’s pairwise tests indicated a difference (*p*_bonf__‑_= 0.012) between the age brackets 25 to 34 years and 55 to 64 years, with median responses of 16 (agree) and 19 (strongly agree), respectively. There was no significant difference between other groups.

**TABLE 5:** Differences in responses to statements among groups.

Variable	Group	*n*	*df*	Statements 1–4				Statement 5				Statement 6				Statement 7				Statement 8			
				Median	IQR	H	*p*	Median	IQR	H	*p*	Median	IQR	H	*p*	Median	IQR	H	*p*	Median	IQR	H	*p*
**Age bracket**	18 to 24	37	5	16	12–18	14.55	0.012	4	3–5	17.489	0.004	3	2–4	19.59	0.001	4	2–5	22.368	< 0.001	3	2–4	14.525	0.013
	25 to 34	389		16	12–19			3	2–5			2	1–4			3	2–4			3	2–4		
	35 to 44	163		17	10.5–20			3	2–5			2	1–4			3	1.5–5			3	2–4.5		
	45 to 54	161		18	12–20			3	2–4			2	1–4			2	1–4			3	2–5		
	55 to 64	127		19	14–20			3	2–4			2	1–3			2	1–3			4	3–5		
	Over 65	123		18	12.5–20			3	2–4			2	1–3			2	1–4			4	2–5		
**Sector**	Public	398	2	16	12–19	13.151	0.001	3	2–4	3.864	0.145	2	1–4	5.715	0.057	3	2–4	4.083	0.13	2	2–4	5.215	0.074
	Private	400		18	13–20			3	2–4			2	1–4			2	1–4			3	2–5		
	Both	104		17	8–19.25			3	2–5			2.5	1–5			3	1–4			3	2–5		
**Level of MP**	HOD	27	9	17	13–20	21.089	0.012	3	2–4.5	28.947	< 0.001	1	1–3	34.795	< 0.001	3	1.5–4	28.411	< 0.001	3	1.5–4	25.402	0.003
	Consultant	214		19	15–20			2	1–4			2	1–3			2	1–3			4	2.5–5		
	Registrar	65		17	11–20			4	2–5			2	1–4			3	2–5			4	3–5		
	MO	243		17	10–20			3	2–4			3	1–4			2	1–4			3	2–4		
	CSMO	33		18	13–20			3	2–4			2	2–4			2	2–4			3	2–4		
	Intern	65		16	13–18			3	2–4			2	1–4			3	2–4			3	2–4		
	Student	39		16	8–19			4	3–5			3	2–5			4	2–5			3	2–4		
	Retired	49		18	12–20			3	2–4			2	1–3			3	2–4			3	2–4		
	GP	55		17	11–20			3	2–5			2	1–3.5			2	1–4			3	2–4		
	Other	42		18	14–20			3	2–5			2	1–4.75			2.5	1–5			3	2–5		
Province*	EC	52	8	18	11–20	18.149	0.02	3.5	2–5	1.938	0.983	2	1–4	19.049	0.015	2	1–4	6.009	0.646	3	2–5	14.005	0.082
	FS	26		17	11.25–19			3	2–5			2	2–3			3	1.25–4			3	2–4		
	GP	330		18	13–20			3	2–4.75			2	1–4			2	1–4			3	2–5		
	KZN	249		16	13–19			3	2–4			3	2–4			3	2–4			3	2–4		
	LP	11		9	5–12.5			3	1–5			3	1–5			3	1.5–3.5			3	1–3		
	MP	23		16	8–19			3	2–5			3	1.5–5			2	1–3.5			4	1.5–5		
	NW	22		20	12.25–20			3	2–4.75			3	1.25–4.75			2	1–3			4	2.25–5		
	NC	9		16	8–19			2	2–5			2	2–5			2	2–3			2	1–4		
	WC	180		18	13–20			3	2–4			2	1–3.25			2	1–4			4	2–5		

Statistical tests: Kruskal Wallis.

* , Students and retired healthcare workers not included.

IQR, interquartile range; CSMO, community service medical officer; *df*, degrees of freedom; EC, Eastern Cape; FS, Free State; GP, Gauteng (in context of province) or general practitioner/family physician (in context of MP level); HOD, head of department; KZN, KwaZulu-Natal; LP, Limpopo; MO, medical officer; MP, Mpumalanga (in context of province) or medical practitioner (in context of HCW type; NW, North West; NC, Northern Cape; WC, Western Cape.

Age brackets were subsequently grouped into a younger (< 45 years) and older (≥ 45 years) group, and responses between the two groups were compared (see Table 6). Similar distribution plots were observed between the two groups, and a Mann–Whitney *U* test indicated a difference (*p* = 0.001) in the medians of the two groups, with the younger age group agreeing to a lesser degree (median = 17) to the older group (median = 18), although both groups strongly agreed that C19VPs are a good idea.

**TABLE 6:** Differences in responses between groups.

Variable	Group	*n*	Statements 1–4				Statement 5				Statement 6				Statement 7				Statement 8			
			Median	IQR	*U*	*p*	Median	IQR	*U*	*p*	Median	IQR	*U*	*p*	Median	IQR	*U*	*p*	Median	IQR	*U*	*p*
Age	< 45 years	589	17	12–20	135 396	0.001	3	2–5	106 820	0.001	2	1–4	107 183	0.002	3	2–4	103 427	< 0.001	3	2–4	135 579	< 0.001
	≥ 45 years	411	18	13–20			3	2–4			2	1–3			2	1–4			4	2–5		
Residency	SA citizen	964	17	12–20	18 435	0.518	3	2–4.25	20 568	0.053	2	1–4	18 564	0.464	2.5	1–4	17 593	0.885	3	2–4	17 816	0.781
	Non-SA citizen	36	19	10–20			4	2–5			2.5	1–4.25			2.5	1.75–4.25			3	2–5		
Practitioner type	MP	832	17	12–20	74 810	0.143	3	2–4	61 241	0.01	3	1–4	59 445	0.002	2	1–4	59 614	0.002	3	2–5	71 535	0.622
	Non-MP	168	17	12–19			3	2–5			3	2–4			3	2–5			3	2–4		
Studying	Students	42	15.5	8.5–19	23 201	0.087	3.5	3–5	16 201	0.029	3	2–4.75	15 070	0.005	4	2–5	15 824	0.016	3	2–4	22 622	0.162
	Non-students	958	17	12–20			3	2–5			2	1–4			2	1–4			3	2–4.75		
Proportion of provincial population vaccinated*	Highest	599	18	12–20	97 451	0.065	3	2–5	87 898	0.43	2	1–4	76 066	< 0.001	2	1–4	86 520	0.241	3	2–5	94 177	0.342
	Lowest	303	17	12–20			3	2–4			3	2–4			3	2–4			3	2–4		

* , Statistical tests: Mann-Whitney *U*.

* , Students and retired healthcare workers not included. Highest proportion at the time of survey = WC, EC, LP, FS & GP; lowest = KZN, MP, NW & NC (COVID-19 SA 2020).

EC, Eastern Cape; FS, Free State; GP, Gauteng; IQR, interquartile range; KZN, KwaZulu-Natal; LP, Limpopo; MP, Mpumalanga (in context of province) or medical practitioner (in context of HCW type); NW, North West; NC, Northern Cape; SA, South Africa; WC, Western Cape.

There was a statistically significant difference (*p* = 0.001) between responses between different sectors of practice (see Table 5). Dunn’s post-hoc analyses provided strong evidence (*p*_bonf_ < 0.001) for a difference between responses from HCWs working in the private sector (median = 18) and those in the public sector (median = 16), with private sector HCWs more likely to agree that C19VPs are a good idea. There was also a difference (*p*_bonf_ = 0.042) between responses from HCWs working in both public and private (median = 17) compared to HCWs working only in the private sector, with private sector HCWs also more likely to agree than those working in both sectors.

Medical practitioners at different levels of practice responded differently to grouped questions 1–4 (*p* = 0.012, see Table 5). Dunn’s post-hoc analyses indicated that the differences were between consultants (median = 19) and medical officers (MOs) (median = 17), although all levels of practice had median responses in agreement that C19VPs are a good idea.

When analysing the results from different provinces for responses regarding whether C19VPs are a good idea, there was a difference found (*p* = 0.02, see Table 5). Importantly, the distributions for these results did not all follow the same shape or scale, and the sample sizes for some of the provinces were small, so this result should be treated with reserve. Dunn’s pairwise tests resulted in significant results when comparing LP to WC (*p*_bonf_ = 0.018), NW (*p*_bonf_ = 0.025), GP (*p*_bonf_ = 0.023) and EC (*p*_bonf_ = 0.046), with LP appearing to be a statistical outlier with the lowest median response (9: disagree).

There were, however, no differences found in responses to grouped statements 1–4 using the Mann-Whitney U test to compare groups of provinces with the most versus fewest confirmed cases of COVID-19 (*p* = 0.304), the most versus the fewest deaths (*p* = 0.097), or the highest versus lowest proportion of population vaccinated at the time of the survey (*p* = 0.065; see Table 6).

When comparing responses to grouped statements 1–4 by respondents’ residency status, there was no difference in responses between non-SA and SA citizens (*p* = 0.518), as shown in Table 6. Additionally, Mann–Whitney U tests (see Table 6) comparing differences in responses for grouped statements 1–4 revealed no difference when comparing MPs to non-MP HCWs (*p* = 0.143), nor any difference comparing students to non-students (i.e. working or retired HCWs: *p* = 0.087).

### Statement 5: Are COVID-19 vaccination passports unethical, considering variations in vaccine access?

Responses for statement number 5 were widely spread as seen in Figure 1, with a median ‘neutral’ response.

There was a significant difference (*p* = 0.004) between responses in different age brackets, shown in Table 5. Dunn’s post-hoc tests found a difference between 55 and 64 age bracket (median = 3) and the 18–24 age bracket (median = 4, *p* = 0.049), as well as between the 55–64 age bracket and the 25–34 age bracket (median = 3, *p* = 0.04). Notably, the distribution plots between the groups were not the same shape – the responses of the 18–24 group and 25–34 group were skewed to the left, with the most frequent response for both groups being ‘strongly agree’, compared to the remaining groups which displayed relatively equal numbers for each of the responses 1 to 5.

**FIGURE 2:**
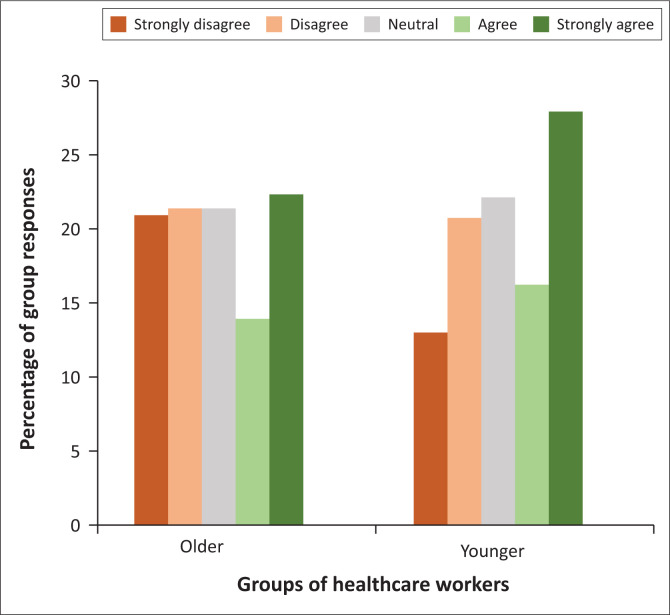
Responses to statement 5 in younger and older healthcare workers.

When age groups were dichotomised into a younger and older group of HCWs, there was a significant difference (*p* = 0.001, see Table 6). However, as noted above, the distribution shape between the groups is not the same (see Figure 2), thus the Mann-Whitney *U* test becomes a test of ranks, not of medians. The younger age group was more likely to agree that C19VPs were unethical due to variations in vaccine access compared to the older age group.

There was a statistically significant difference in the responses to statement 5 between MP (median = 3) and non-MP HCWs (median = 3, see Table 6), with differently-shaped distributions (*p* = 0.01). Non-MPs were more likely to agree that C19VPs were unethical due to variations in vaccine access, although the sample of non-MPs was relatively small. There was also a difference (*p* = 0.029) in responses between students (median = 3.5) and non-students (median = 3), with students more likely to agree with the statement (Table 6).

Responses to this statement were different when comparing MPs practising at different levels (*p* < 0.001, see Table 5). Dunn’s pairwise tests revealed the difference to lie in the responses from consultants (median = 2) compared to registrars (median = 4, *p*_bonf_ = 0.003) and between consultants and students (median = 4, *p*_bonf_ = 0.004).

When comparing responses to statement 5 by respondents’ residency status, there was no difference in responses between non-SA- and SA citizens (*p* = 0.053), as shown in Table 6. There was also no difference between responses to this statement and sector of practice (*p* = 0.145, see Table 5), with a median response of 3 from each sector. Analysing the responses from different provinces also did not yield a statistically significant difference in responses (*p* = 0.983), with a median response of ‘neutral’ for all provinces except for NC with a median response of ‘disagree’ (Table 5).

### Statement 6: Are COVID-19 vaccination passports unethical due to personal, religious or philosophical objections?

Respondents tended to disagree that these factors make C19VPs unethical, with 54.7% of HCWs, the majority being MPs, disagreeing overall.

There was again a significant difference in responses to this statement across age groups using the Kruskal-Wallis test (*p* = 0.001, see Table 5). Post-hoc pairwise testing showed the differences to lie between 18 and 24 (median = 3) and the groups 55–65 (median = 2, *p*_bonf_ = 0.009) and over 65 (median = 2, *p*_bonf_ = 0.044). There was also a difference in ranks between the responses from the 25–34 bracket and 55–64 bracket (*p*_bonf_ = 0.005), although the median response in both groups was ‘disagree’.

When grouped into a younger and older group of HCWs (see Table 6), the responses to statement 6 again varied in their distribution (*p* = 0.002), although the median response in both groups was ‘disagree’, with the younger group having a wider IQR (1–4) in their responses compared to the older group (1–3).

A difference was also found when comparing responses from respondents in different provinces, as shown in Table 5. Dunn’s post-hoc tests provided evidence for differences between KZN (median = 3) and GP (median = 2) and between KZN and WC (median = 2, *p*_bonf_ = 0.025). Healthcare workers in GP and WC were more likely to disagree that personal objections to vaccination make C19VPs unethical, compared to HCWs in KZN.

Notably, as shown in Table 6, there was a statistically significant difference (*p* < 0.001) in responses when comparing provinces grouped into those with the highest proportion of population vaccinated (WC, EC, LP, FS, GP) to the lowest (KZN, MP, NW, NC) at the time of the survey, with the provinces with the higher vaccination proportion disagreeing more than the provinces with the lower vaccination proportion. No differences found using the Mann Whitney U test to compare provinces with the most and fewest confirmed cases of COVID-19 or most and fewest deaths.

A difference was noted in the distribution of responses from MPs (median = 3) compared to non-MPs (median = 3, see Table 6), with the IQR of MPs (1–4) indicating more ‘strongly disagree’ responses compared to the IQR of non-MPs (2–4, *p* = 0.002). There was also a difference (*p* = 0.005) between the responses from students (median = 3) and non-students (median = 2, see Table 6), with non-students being more likely to disagree that C19VPs are unethical due to vaccine hesitancy-related factors.

There was again a difference noted in responses when comparing MPs at different levels of practice (*p* < 0.001, see Table 5). Pairwise testing revealed these differences to occur between students (median = 3) and 3 other groups of practitioners: consultants (median = 2, *p*_bonf_ < 0.001), Head of Departments (HODs) (median = 1; *p*_bonf_ = 0.016), and retired MPs (median = 2, *p*_bonf_ = 0.035). Students were less likely than these groups of senior MPs to disagree with the statement. There was an additional difference (*p*_bonf_ < 0.001) between consultants and MOs (median = 3), with consultants more likely to disagree that personal objections make C19VPs unethical.

No difference was found when comparing SA- to non-SA citizens (*p* = 0.464, see Table 6) or when comparing sector of practice (*p* = 0.057, see Table 5).

### Statement 7: Will poorer people be further disadvantaged by COVID-19 vaccination passports?

Respondents were more likely to disagree that C19VPs will disadvantage the poor, as shown in Table 4 and Figure 1.

Responses differed between age brackets (*p* < 0.001), as shown in Table 5. Post-hoc testing revealed the differences to lie between the 18–24 age bracket (median = 4) and 3 other groups: 45–54 (median = 2, *p*_bonf_ = 0.017), 55–64 (median = 2, *p*_bonf_ = 0.002) and ≥ 65 (median = 2, *p*_bonf_ = 0.01). There was an additional difference (*p*_bonf_ = 0.012) between the 25–34 bracket (median = 3) and the 55–64 bracket. The older age groups were more likely to disagree that poorer people would be disadvantaged by C19VPs compared with their younger colleagues, which was confirmed by the Mann-Whitney *U* test comparing the responses in the ≥ 45 group (median = 2) to the < 45 group (median = 3, *p* < 0.001, see Table 6).

A statistically significant difference was noted when comparing responses to this statement between MP (median = 2) and non-MP HCWs (median = 3, *p* = 0.002, see Table 6). Medical practitioners were more likely to disagree that C19VPs are unethical because poor people will be further disadvantaged, compared to other types of HCW, which may have been affected by the small number of non-MPs in the sample.

A difference in responses was also observed between students (median = 4) and non-students (median = 2), where students were more likely to agree with the statement, while non-students disagreed (*p* = 0.016, see Table 6).

Different levels of MPs responded differently to this statement (*p* < 0.001, see Table 5). Dunn’s post-hoc comparisons revealed the differences were between consultants (median = 2) and students (median = 4, *p*_bonf_ = 0.001) and between consultants and interns (median = 3, *p*_bonf_ = 0.013). Consultants were more likely to disagree, compared to interns (‘neutral) and students (‘agree’).

No differences were observed in responses to this statement between SA- and non-SA citizens (*p* = 0.885, see Table 6), nor between responses from HCWs in different sectors of practice (*p* = 0.13, see Table 5). Additionally, there was no difference in responses from respondents in different provinces (*p* = 0.646, see Table 5).

### Statement 8: Are COVID-19 vaccination passports feasible in SA?

There was wide variation in the responses to this statement (see Figure 1), with the median response being neutral (Table 4).

A difference in responses to the statement was noted between age brackets (*p* = 0.013, see Table 5). The difference was found to occur between the age brackets 25–34 (median = 3) and 55–64 (median = 4, *p*_bonf_ = 0.007), with the 55–64 age group being more likely to agree that C19VPs are feasible in SA. This difference in responses between younger and older HCWs was also demonstrated when comparing the ≥ 45 age group (median = 4) to the < 45 age group (median = 3), with the older age group more likely to agree with feasibility (*p* < 0.001, see Table 6).

There was a difference (*p* = 0.003) noted when comparing responses from different levels of MP (see Table 5). The post-hoc testing revealed this difference to lie between consultants (median = 4) and MOs (median = 3, *p*_bonf_ = 0.002) and consultants and registrars (median = 3, *p*_bonf_ = 0.028). Consultants were more likely to agree with feasibility compared to MOs and registrars. No other differences existed between other groups.

There was no difference in opinion on feasibility of C19VPs in SA when comparing SA- to non-SA citizens (*p* = 0.781, see Table 6), sector of practice (*p* = 0.074, see Table 5), province (*p* = 0.082, see Table 5), MPs to other HCWs (*p* = 0.622, see Table 6), or students to non-students (*p* = 0.162, see Table 6).

Ordinal logistic regression analyses showed that HCWs’ opinion on feasibility could be somewhat predicted according to whether they thought C19VPs are a good idea. If respondents thought C19VPs were a good idea, there was a 59.7% chance they would respond that they are feasible; whereas disagreeing that C19VPs are a good idea yielded an 82.3% chance of respondents saying they are not feasible, with a McFadden’s pseudo R-square value of 0.188. Ordinal logistic regression analyses between other responses yielded models with lack-of-fit (goodness of fit indicators *p* < 0.05) and therefore could not be used to predict outcomes.

## Discussion

The perspectives of HCWs in SA on C19VPs varied. A wide range of HCWs responded to the survey, with equal numbers from the private and public sectors, but with the majority being younger HCWs under the age of 45, three-quarters coming from only three provinces, GP, KZN and WC and with a significant majority being MPs. This was most likely a result of the convenience sampling used, with dissemination of the survey via social media and presents a limitation in the generalisability of the findings of this study.

Almost three-quarters of respondents in SA responded that C19VPs are a good idea – significantly more than previous studies have found when assessing support for immunity passports among the general public (Hall & Studdert 2021; Largent et al. 2020). This may be due to HCWs’ direct involvement in the frontline of the pandemic, which has resulted in significant harm to HCWs as a result of their increased risk of being infected with COVID-19, leading to more than 115 000 HCW deaths, in addition to the pandemic’s significant effects on HCW mental health (Pappa et al. 2020; World Health Organization 2021).

The older demographic of HCWs tended to agree more strongly that C19VPs were a good idea compared to the younger demographic and was more likely to think that implementation of C19VPs in SA is feasible; responses which may be influenced by many factors, including their increased risk of morbidity and mortality associated with COVID-19 infection.

Healthcare workers in private practice were more likely to agree that C19VPs are a good idea compared to their state practice counterparts, perhaps because they have experienced the economic impact of the pandemic first-hand on their practices’ reduced patient loads and finances (Van den Heever & Dasoo 2021), although sector of practice had no effect on HCWs opinions regarding ethical issues or feasibility.

In SA, HCWs were some of the first to be vaccinated through the Sisonke Programme, a collaboration between the National Department of Health, South African Medical Research Council, CAPRISA, Desmond Tutu Health Foundation, Janssen and Johnson and Johnson (South African Medical Research Council 2021). The open-label phase 3b trial vaccinated nearly 480 000 HCWs with Janssen’s Ad26.COV2.S between 17 February and 17 May 2021 and has proven to provide durable and effective protection against variants of concern, including the Delta variant (Gray 2021; Keeton 2021).

The national vaccine rollout has entered phase 3, with all adults now being eligible to receive the vaccination (South African Government 2021a). At the time of the survey, the vaccination was not yet available to all groups, and younger HCWs and students were more likely to think that C19VPs are unethical due to vaccine access variations. These young HCWs may have had peers who have had difficulty accessing vaccination due to the phased roll-out, or may have even had difficulty in accessing vaccinations themselves, in the case of student HCWs who may have not been vaccinated in the Sisonke Programme.

The vaccine rollout in SA is accessible free of charge to everyone, but questions have been raised about the fairness of the English-only, digital EVDS, which initially required internet access and an appropriate device to register for vaccination (South African Government 2021b). Access to vaccination sites for people living in rural and remote areas has also raised concerns (Bloomberg 2021). Respondents in this study mostly disagreed that poorer people will be further disadvantaged by vaccine certification, with the exception of younger HCWs and students, with other categories of HCW besides MPs remaining neutral, although these represented a minority in the study.

The majority of respondents across all groups disagreed that individuals’ personal, religious, or philosophical objection to vaccination make vaccine certification unethical. The younger demographic, students and non-MP HCWs disagreed to a lesser degree than their comparator groups. This is in keeping with findings from the HSRC, which showed increasing levels of vaccine hesitancy with decreasing age (Runciman et al. 2021). Research in other countries has also revealed relatively high levels of vaccine hesitancy among certain HCW groups: in the USA, up to a quarter of medical students were hesitant about COVID-19 vaccinations (Lucia, Kelekar & Afonso 2021), and in France, older HCWs and MPs (compared with nurses) were more likely to readily accept vaccination (Gagneux-Brunon et al. 2021).

The WHO named vaccine hesitancy as one of the top 10 threats to global health in 2019 due to its contribution to the resurgence of vaccine-preventable diseases such as measles and said that ‘health workers, especially those in communities, remain the most trusted advisor and influencer of vaccination decisions’ (World Health Organization 2019), which has remained true for COVID-19 vaccinations (Reiter, Pennell & Katz 2020).

The authors did not specifically enquire after HCW vaccination status in their survey; however, respondents from provinces with the highest vaccination proportion were more likely to disagree that C19VPs are unethical due to individual objections to vaccination, compared to their counterparts in provinces with a lower proportion of the population vaccinated.

The question of the feasibility of C19VPs in SA elicited varying responses from HCWs, with those who thought it was a good idea believing it to be feasible. Despite doubts, the South African COVID-19 Vaccine Certificate has been launched and is set to be used for a variety of purposes (Daniel 2021).

This study explored the perspectives of HCWs in SA around C19VPs but had certain limitations. The convenience sampling technique used in this study did not obtain a sample representative of the different subsets of HCWs, with the proportion of MPs in the sample far exceeding that expected in the South African HCW population. It is expected that those who feel strongly about COVID-19 vaccinations (either positively or negatively) would have been more likely to take interest and respond to the survey. The rapidly evolving nature of the pandemic means that HCW responses may also evolve over time – the authors captured their perspectives during the third wave of infections, before vaccination was available to all adults and before implementation of the South African COVID-19 Vaccine Certificate. Additionally, Likert-type responses are known to be subject to distortion through several mechanisms. Central tendency bias arises where individuals are more likely to avoid extreme answers – this was mitigated by dichotomisation of results for statistical analysis. Acquiescence bias is a tendency to agree with statements as presented, which the authors attempted to avoid by presenting specific statements and then grouping the results, rather than broad opinions on whether it is a good or bad idea.

## Conclusion

In conclusion, most HCWs sampled (the majority being MPs) thought that C19VPs are a good idea, with little regard to vaccine hesitancy in certain groups, with some concerns regarding the ethical issues of vaccine access, and uncertainty about feasibility. To the authors’ knowledge, this is the first survey data published on the perspectives of HCWs on C19VPs and the associated ethical issues. Healthcare workers are trusted influencers of vaccination decisions, and their opinion on vaccination certificates may also influence the public’s perception and acceptance thereof. Future research into vaccine hesitancy itself in SA, especially in HCWs and students, would be valuable, as well as further research into the efficacy of C19VPs in reducing the transmission of COVID-19, and any beneficial effects they may have on individuals’ mental health or on economies of countries in which they have facilitated the lifting of restrictions.
